# Gastrointestinal symptom profiles after resection of esophageal or gastric cancer

**DOI:** 10.1007/s00520-026-11293-7

**Published:** 2026-10-07

**Authors:** Yipei Lee, Dimitris Katsimpokis, Felice N. van Erning, Grard A. P. Nieuwenhuijzen, Hanneke W. M. van Laarhoven, Suzanne S. Gisbertz, Bastiaan R. Klarenbeek, Paul M. Jeene, Roos E. Pouw, Loes Noteboom, Rob H. A. Verhoeven, Pauline A. J. Vissers

**Affiliations:** 1 Department of Research and Development, Netherlands Comprehensive Cancer Organization (IKNL), Rijnkade 5, 3511 LC Utrecht, The Netherlands; 2 Department of Medical Oncology, Amsterdam UMC Location University of Amsterdam, De Boelelaan 1117, 1081 HV Amsterdam, The Netherlands; 3 Cancer Center Amsterdam, Cancer Treatment and Quality of Life, De Boelelaan 1117, 1081 HV Amsterdam, The Netherlands; 4 Department of Surgery, Amsterdam UMC Location University of Amsterdam, De Boelelaan 1117, 1081 HV Amsterdam, The Netherlands; 5 Department of Surgery, Radboud University Medical Center, Geert Grooteplein Zuid 10, 6525 GA Nijmegen, The Netherlands; 6 Department of Surgery, Catharina Hospital, Michelangelolaan 2, 5623 EJ Eindhoven, The Netherlands; 7 Radiotherapiegroep, Nico Bolkesteinlaan 85, 7416 SE Deventer, The Netherlands; 8 Department of Gastroenterology and Hepatology, University Medical Center Utrecht, Heidelberglaan 100, 3584 CX Utrecht, The Netherlands; 9 Department of Medical Informatics, Amsterdam UMC, Amsterdam, The Netherlands

**Keywords:** Esophagogastric cancer, Esophagectomy, Gastrectomy, Gastrointestinal symptoms, Symptom profiles, Longitudinal study

## Abstract

**Purpose:**

Persistent gastrointestinal symptoms are common after esophagectomy or gastrectomy, which frequently co-occur and significantly decrease quality of life. This study aimed to identify gastrointestinal symptom profiles over the first-year post-surgery and to explore changes over time.

**Methods:**

Patients diagnosed between 2013 and 2024 who underwent esophagectomy or gastrectomy for nonmetastatic esophageal or gastric cancer were selected. Longitudinal self-reported gastrointestinal symptom data were linked to patient and tumor characteristics. K-means clustering based on symptom severity scores was performed at baseline and at every three months up to 12 months postoperatively. Generalized Linear Mixed Models assessed associations between patient characteristics and symptom profiles.

**Results:**

Among 1462 patients who underwent esophagectomy and 434 who underwent gastrectomy, three symptom severity profiles, mild, moderate, and severe, were identified for each period. These profiles reflected increasing symptom severity rather than distinct symptom patterns. The mild symptom profile was the most common (esophagectomy: 42.5 ~ 60.5%; gastrectomy: 53.9 ~ 73.5% across time periods). Most patients remained in the same profile over time. Amongst others, female sex (esophagectomy: OR = 1.67; 95% CI:1.30–2.14, gastrectomy: OR = 1.54; 95% CI:1.07–2.23) and receiving neoadjuvant chemotherapy (esophagectomy: OR = 1.91; 95% CI:1.22–2.99, gastrectomy: OR = 2.39; 95% CI:1.55–3.67) were associated with a higher likelihood of moderate/severe symptom profile.

**Conclusions:**

Three stable gastrointestinal symptom severity profiles were identified during the first postoperative year, supporting risk-stratified monitoring after upper gastrointestinal cancer surgery. Early identification of patients at risk for persistent moderate-to-severe gastrointestinal symptoms may facilitate timely supportive care and improve survivorship outcomes.

**Supplementary Information:**

The online version contains supplementary material available at https://doi.org/10.1007/s00520-026-11293-7.

## Introduction

Globally, 1.49 million new cases of esophageal and gastric cancer were diagnosed in 2022 [1]. While resection with (neo)adjuvant chemo(radio)therapy offers a potential for cure [2, 3], it frequently leads to significant gastrointestinal dysfunction due to the anatomical changes of the gastroesophageal tract. More than one-third of patients experience persistent gastrointestinal symptoms at 9–12 months after surgery, around 20% and 15% of patients report two or more severe gastrointestinal symptoms following esophagectomy or gastrectomy, respectively [4]. These gastrointestinal symptoms include eating restrictions, loss of taste, reflux, diarrhea, and appetite loss [5–7], which can significantly decrease patients’ quality of life [4, 8, 9].

Previous research revealed that gastrointestinal symptoms after resection frequently co-occur, suggesting potential patterns or clusters of symptoms [9]. Moreover, literature also shows that having two or more gastrointestinal symptoms was associated with clinically relevant deteriorations in health-related quality of life (HRQoL), functioning, work productivity and impairment in daily activities [4]. Work productivity impairment was reported to be 29.3% higher for patients with more than two gastrointestinal symptoms after esophagectomy compared with those without gastrointestinal symptoms. Meanwhile, impairment in daily activities was reported to be 19.4% and 21.8% higher for patients with two or more gastrointestinal symptoms after esophagectomy or gastrectomy, respectively [4].

To further understand the clustering of symptoms for better symptom management, traditional approaches like exploratory factor analysis (EFA) have been widely used to identify the dominant factors for each symptom profile [10–13]. Psychological symptom cluster, chemotherapy-related symptom cluster, and gastrointestinal symptom cluster were frequently reported for gastrointestinal cancer patients [11, 12]. However, few studies have clarified the long‑term outcomes of gastrointestinal symptom patterns, and cross‑sectional clustering approaches may oversimplify the complex, dynamic evolution of symptoms observed in practice. Despite evidence of long-term HRQoL impairments and co-occurrence of symptoms after esophagectomy and gastrectomy, little is known about how they evolve over time. Examining symptom profiles is crucial, as they may serve as stronger predictors of functional outcomes and HRQoL compared to single symptoms, thereby offering a more comprehensive framework for understanding patients’ long-term outcomes and for targeting symptom management. This study aims to identify and track gastrointestinal symptom profiles over the first year post-surgery in patients with esophageal or gastric cancer, providing an exploratory foundation for future studies and clinical validation.

## Material and methods

### Data collection

Longitudinal data for this study was obtained from the Prospective Observational Cohort study of Oesophageal-gastric Cancer Patients (POCOP), which is routinely linked to the Netherlands Cancer Registry (NCR). POCOP is a nationwide registry, collecting patient-reported outcomes [14]. Previous research has demonstrated that patients enrolled in POCOP are broadly representative of the overall Dutch population of individuals diagnosed with esophageal or gastric cancer, supporting the generalizability of the findings [15]. Patients can enroll at any stage of their disease or treatment and receive questionnaires at predefined intervals (e.g. baseline, 3, 6, 9, 12, 18, 24 months and annually thereafter). Patient and tumor characteristics and treatment data are retrieved through linkage with the NCR, a population-based registry that systematically records all newly diagnosed malignancies in the Netherlands.

### Study population

Patients with nonmetastatic esophageal or gastric cancer (cM0) diagnosed between 2013 and 2024 were selected, provided that their registration in the NCR was complete and they had undergone an esophagectomy or gastrectomy. Patients were required to complete at least two POCOP questionnaires with no missing data on gastrointestinal symptom scales within the follow-up periods. These intervals were defined as the pre-resection phase (either before, during or after neoadjuvant therapy): baseline, and post-resection periods: 0–3, 3–6, 6–9, and 9–12 months.

### Patient, tumor, and treatment characteristics

The following patient characteristics were retrieved from the NCR: patient characteristics (sex, age, ASA classification [16], number of comorbidities), tumor factors (clinical T and N, histology), treatment factors (surgery type, receipt of (neo)adjuvant chemo(radio)therapy), and prolonged hospital stay after surgery, defined as the 75th percentile: > 12 days for esophagectomy and > 8 days for gastrectomy, as had been done previously [4].

Numbers of comorbidities are documented using a modified Charlson Comorbidity Index. Staging of the primary tumor was conducted using the Union for International Cancer Control’s tumor-node-metastasis classification, applicable to the diagnosis period (7th edition for 2013–2016 and 8th edition for 2017–2024) [17, 18]. The tumor’s anatomical location was classified according to the International Classification of Disease—Oncology (ICD-O–3) [19].

### Gastrointestinal symptoms

Gastrointestinal symptoms were retrieved from scales of both the European Organization for Research and Treatment of Cancer Quality of Life Questionnaire Core 30 (EORTC QLQ-C30) and the EORTC QLQ Oesophageal–Gastric Cancer Module 25 (EORTC QLQ-OG25) as collected within POCOP. The following gastrointestinal symptoms were included in this study from the EORTC QLQ-C30: the nausea/vomiting scale (two items) and single items constipation, diarrhea, appetite loss, and the scales from the EORTC QLQ-OG25: dysphagia (three items), eating restrictions (four items), reflux (two items), odynophagia (two items), pain and discomfort (two items), and single items dry mouth, choked when swallowing, trouble with taste, trouble with coughing, trouble swallowing saliva, and trouble talking. A detailed list of the questions used to evaluate these specific items and scales is available in Supplementary Table 1. All items are scored on a 4-point Likert scale and were linearly transformed to a score between 0 and 100 according to the EORTC scoring guidelines.

### Statistical analyses

Baseline characteristics were retrieved separately for patients who underwent esophagectomy and gastrectomy. The scaled scores for the selected gastrointestinal symptoms at each period were used as the features for k-means clustering, which was performed separately at each period. To determine the optimal number of clusters (k), candidate solutions ranging from k = 1 to 10 were evaluated using the Elbow method [20]. The Elbow method calculates the Within-Cluster Sum of Squares (WCSS) for different values of k and identifies the point at which the rate of decrease in WCSS begins to slow. The final number of clusters was selected based on the Elbow method together with the interpretability of the resulting symptom profiles. To evaluate the clustering results, Silhouette scores, ranging between −1 and 1, were used to assess the compactness and separation of clusters, with scores closer to 1 indicating better clustering.

The patients who underwent an esophagectomy or gastrectomy were assigned to their symptom profiles at each period based on the severity scores of selected gastrointestinal symptoms. Each profile centroid represents the average symptom pattern of patients assigned to that profile. To identify the overall trend of symptom severity, a mean score was calculated for each cluster by averaging the symptom severity scores of the centroid. We tracked changes in patients’ symptom profile membership between each pair of consecutive three-month assessment periods, including only patients with questionnaire data available at both time points of each interval. To assess which patient, tumor and treatment characteristics were associated with the symptom profiles, we fitted generalized linear mixed models (GLMMs) for a binary outcome using a logistic function. The dependent variable was symptom severity profile, dichotomized as “mild” vs. “moderate/severe” based on the clustering results. Fixed effects included sex, age, ASA classification, number of comorbidities, clinical T and N, histology, surgery type, receipt of (neo)adjuvant chemo(radio)therapy, included as time-dependent variables (Patients were classified as untreated (0) until the start of therapy and as treated (1) thereafter.), prolonged hospital stay, and assessment period. A random intercept at the patient level was included to account for within-patient correlation of repeated observations. The analyses were conducted with Python programming language, version 3.12.4. GLMMs were built by R programming language, version 4.5.1.

## Results

### Study population

The total study population included 1896 patients of whom 1462 (77.1%) underwent an esophagectomy and 434 (22.9%) gastrectomy. Most of the patients completed the questionnaire before the resection (95.5% and 92.4% for esophagectomy and gastrectomy, respectively, Table 1). Among patients whose baseline questionnaire was completed on or after initiation of neoadjuvant treatment, the median time from treatment initiation to questionnaire completion was 17 days (IQR 7–48 days) for patients who underwent esophagectomy (n = 741) and 23 days (IQR 7–60 days) for patients who underwent gastrectomy (n = 129). Most patients in both groups were aged 60–74 years, male, had an ASA score of II, and no comorbidity. Among patients who underwent an esophagectomy, 91% received neoadjuvant chemoradiation, whereas 64.5% of the patients who underwent a gastrectomy received neoadjuvant chemotherapy and 36.9% adjuvant chemotherapy or immunotherapy.

**Table 1:** Baseline characteristics of patients who underwent an esophagectomy or gastrectomy

	Esophagectomy		Gastrectomy	
	N	%	N	%
	1462	77.1	434	22.9
Age				
< 60 years	297	20.3	79	18.2
60–74 years	1009	69.0	237	54.6
≧75 years	156	10.7	118	27.2
Sex				
Male	1183	80.9	277	63.8
Female	279	19.1	157	36.2
ASA classification				
ASA I	80	5.5	11	2.5
ASA II	904	61.8	245	56.5
ASA ≧III	423	28.9	139	32.0
Unknown	55	3.8	39	9.0
Number of comorbidities				
No comorbidity	787	53.8	222	51.2
1 comorbidity	450	30.8	131	30.2
2 or more comorbidities	179	12.2	53	12.2
unknown	46	3.1	28	6.5
Histology				
Squamous cell carcinoma	217	14.8	-	-
Adenocarcinoma—intestinal	863	59.0	226	52.1
Adenocarcinoma—diffuse	174	11.9	139	32.0
Adenocarcinoma—other	86	5.9	45	10.4
Unknown	122	8.3	24	5.5
cT				
cT1–2	414	28.3	112	25.8
cT3–4	1005	68.7	286	65.9
cTX	43	2.9	36	8.3
cN				
cN0	632	43.2	248	57.1
cN +	819	56.0	184	42.4
cNX	11	0.8	2	0.5
Type of resection				
Transhiatal esophagectomy	147	10.1	-	-
Transthoracic esophagectomy—Ivor Lewis	964	65.9	-	-
Transthoracic esophagectomy—McKeown	306	20.9	-	-
Transthoracic esophagectomy—Others or Unknown	21	1.4	-	-
Transcervical esophagectomy—MICE	24	1.6	-	-
Subtotal gastrectomy	-	-	232	53.5
Total gastrectomy	-	-	202	46.5
Neoadjuvant chemotherapy				
No	1361	93.1	154	35.5
Yes	101	6.9	280	64.5
Neoadjuvant chemoradiotherapy				
No	129	8.8	347	80.0
Yes	1333	91.2	87	20.0
Adjuvant chemotherapy or immunotherapy				
No	1189	81.3	274	63.1
Yes	273	18.7	160	36.9
Prolonged hospital stay				
Unknown	63	4.3	20	4.6
No	1059	72.4	313	72.1
Yes	340	23.3	101	23.3
Completed questionnaires				
Baseline (before resection)	1396	95.5	401	92.4
0–3 months after resection	1026	70.2	309	71.2
3–6 months after resection	1112	76.1	332	76.5
6–9 months after resection	1013	69.3	277	63.8
9–12 months after resection	528	36.1	169	38.9

### Symptom profiles across assessment periods

K-means clustering identified a three-profile solution (k = 3) at each period. The corresponding Elbow plots are presented in Supplementary Fig. 1. Because the resulting profiles primarily reflected a gradual increase in overall symptom severity rather than distinct patterns of specific symptom co-occurrence, they were interpreted as patient subgroups with relatively mild, moderate, and severe symptom profiles. The profile-specific centroids, representing the average symptom severity pattern within each profile, are presented in Supplementary Table 2. Supplementary Fig. 2 shows the mean symptom scores with corresponding standard deviations for the mild, moderate, and severe profiles across assessment periods, demonstrating that the profiles remained clearly ordered in relative symptom severity despite shifts in absolute mean scores over time.

For patients who underwent an esophagectomy, Silhouette scores across assessment periods were between 0.12 and 0.21. For patients who underwent a gastrectomy, the Silhouette score for each profile was between 0.19 and 0.36. Regarding profile size across all periods, “mild symptoms” profiles accounted for the largest proportion of patients for both esophagectomy and gastrectomy (esophagectomy: 42.5 ~ 60.5%, gastrectomy: 53.9 ~ 73.5%), followed by “moderate symptoms” (esophagectomy: 31.5 ~ 42.1%, gastrectomy: 24.3 ~ 32.5%), with “severe symptoms” profiles (esophagectomy: 8.0 ~ 15.4%, gastrectomy: 2.3 ~ 15.7%) having the smallest size. Most of the mean scores of each symptom profile for patients who underwent esophagectomy or gastrectomy showed an increase at 0–3 months after resection and a stable decrease thereafter. The only exception was the slight increase in mean scores from 3–6 to 6–9 months after gastrectomy, with 5.9 vs 6.1, 18.9 vs 25.8 and 38.5 vs 58.5 for mild, moderate, and severe profiles, respectively. The scores went down again at 9–12 months after resection (Supplementary Table 2).

**Fig. 1:**
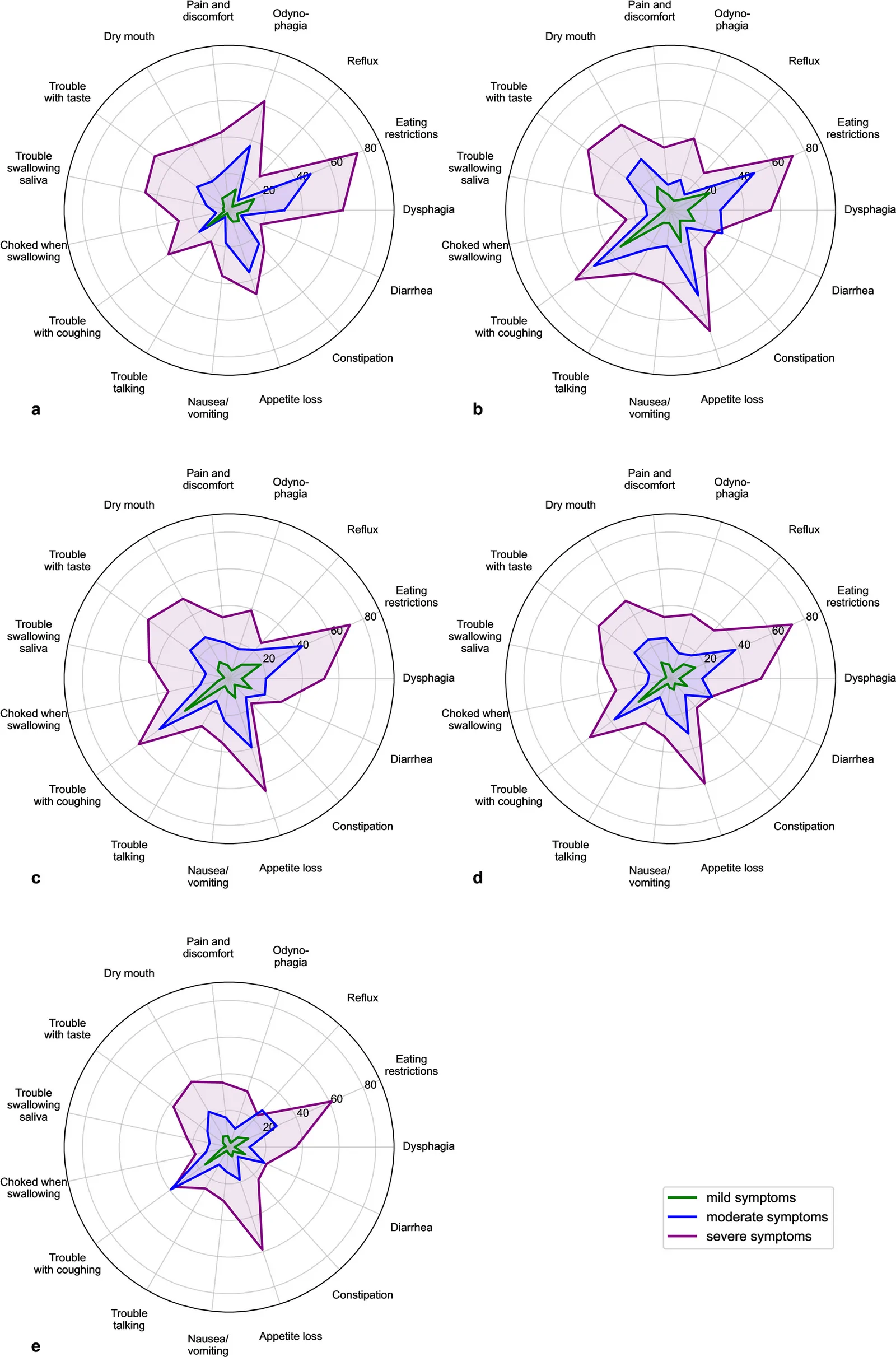
**(a-e)** Symptom patterns of profile-specific centroids in each period for patients who underwent esophagectomy. 1a: baseline (before resection); 1b: 0–3 months after resection; 1c: 3–6 months after resection; 1d: 6–9 months after resection; 1e: 9–12 months after resection

Regarding the symptom patterns, most frequently reported gastrointestinal symptoms were eating restrictions, dysphagia and appetite loss across all time periods for patients after esophagectomy (Fig. 1a-e). Most of the symptom scores followed the order of mild, moderate, and severe profiles with similar patterns. At 9–12 months after resection, patients in the moderate symptom profile reported slightly higher scores for reflux and coughing problems than those in the severe profile (Fig. 1e). Symptoms including dry mouth and trouble with coughing had higher scores after esophagectomy compared to baseline, with the shapes of the symptom patterns being similar at 0–3, 3–6, and 6–9 months after resection (Fig. 1b-d). At 9–12 months after resection (Fig. 1e), trouble with taste, dry mouth, pain and discomfort, odynophagia, eating restrictions, dysphagia, appetite loss, and trouble with coughing remained the frequently reported symptoms, especially for patients in the severe symptom profile.

**Fig. 2:**
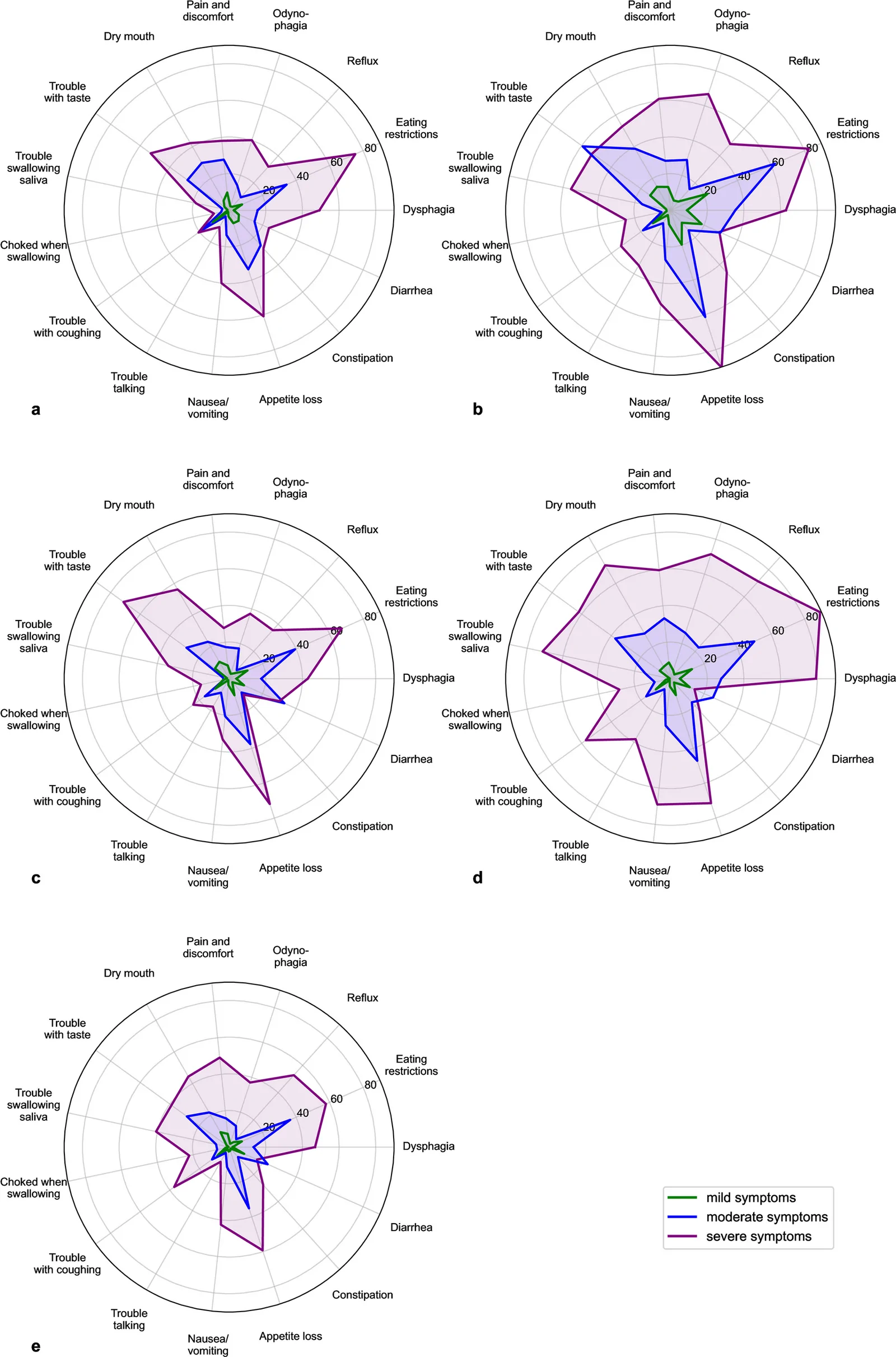
**(a-e)** Symptom patterns of profile-specific centroids in each period for patients who underwent gastrectomy. 2a: baseline (before resection); 2b: 0–3 months after resection; 2c: 3–6 months after resection; 2d: 6–9 months after resection; 2e: 9–12 months after resection

Unlike patients after esophagectomy, the symptom patterns after gastrectomy differed between mild, moderate and severe symptom profiles across assessment periods (Fig. 2a-e). At 0–3 months after resection (Fig. 2b), patients in the severe symptom profile reported higher scores for dysphagia, eating restrictions, appetite loss, pain and discomfort, whereas only trouble with taste was more common in the moderate profile compared to the severe one. Diarrhea was observed more in moderate symptom profiles at 3–6, 6–9, and 9–12 months after resection (Fig. 2c-e). The difference between symptom patterns became more pronounced 6 months after resection (Fig. 2d-e). In the moderate profile, eating restrictions, diarrhea, appetite loss, trouble with taste, and dry mouth were more frequently reported, particularly at 9–12 months after resection (Fig. 2e). In contrast, the severe profile showed higher scores for a broader range of symptoms at both 6–9 and 9–12 months, including dysphagia, nausea/vomiting, coughing, trouble swallowing saliva, and odynophagia, but not diarrhea.

### Changes across symptom profiles over time

**Fig. 3:**
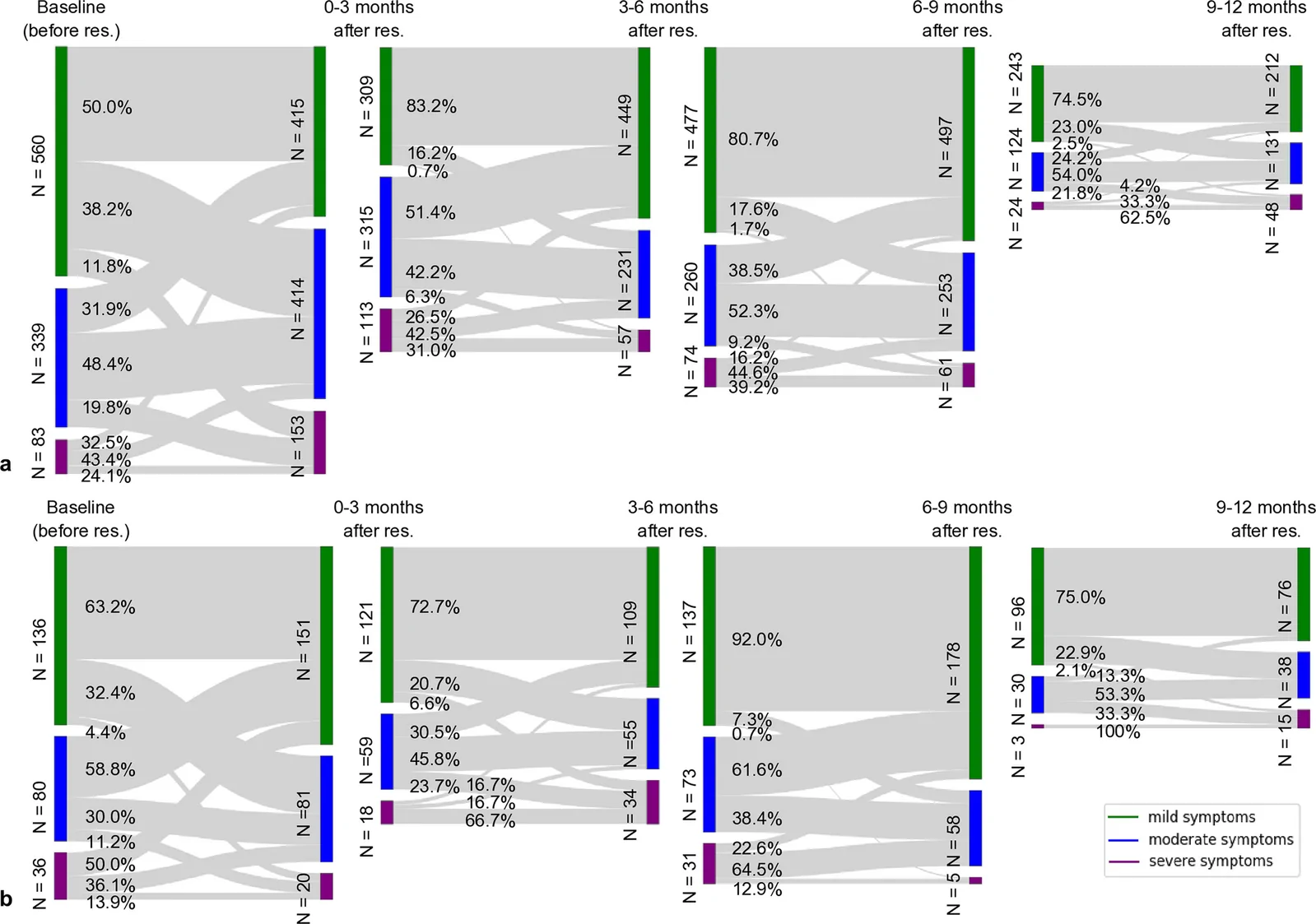
**(a-b)** The percentages of symptom profile transition over time. Different colors indicate each profile as shown in the legend. **3a**: patients who underwent esophagectomy; **3b**: patients who underwent gastrectomy. ***res*** resection (esophagectomy or gastrectomy); green: mild symptom profile; blue: moderate symptom profile; purple: severe symptom profile. (Note: only patients that completed both the start and end of the intervals were shown; due to the large difference in group size, the scales of N for 3a and 3b are not identical.)

For patients who underwent esophagectomy (Fig. 3a), most patients remained in the same symptom severity profile between consecutive assessment periods. Very low percentages, ranging from 0.7% to 2.5% of patients shifted from the mild to the severe symptom profile across postoperative intervals after 3 months. For patients who started in the moderate symptom profile, the proportions who remained in the moderate profile ranged from 42.2% to 54.0% across consecutive intervals. From 0–3 to 3–6 months after resection, more patients shifted to the mild profile (51.4%) compared to those who stayed. The patients who started in the severe symptom profile mostly shifted to the moderate (33.3 to 44.6%).

Similarly, most patients that underwent gastrectomy (Fig. 3b) stayed in the same symptom severity profile over time. Most patients in the mild symptom profile, ranging from 63.2% to 92.0%, did not shift to other profiles across consecutive intervals. Very low percentages of patients, ranging from 0.7% to 6.6%, shifted from the mild to the severe symptom profile across consecutive intervals. From baseline to 0–3 months after resection, around half of patients within the moderate or severe profile shifted to the mild, with 58.8% and 50.0%, respectively. From 3–6 to 6–9 months after resection, 61.6% of patients shifted from moderate to mild, whereas 64.5% of patients shifted from severe to moderate.

### Associations between baseline characteristics and symptom profiles stratified by esophagectomy or gastrectomy

For patients who underwent esophagectomy, female sex (OR 1.67, 95% CI 1.30–2.14), ASA classification ≥ III (OR 1.78, 95% CI 1.12–2.83), transthoracic (McKeown vs transhiatal) surgery (OR 1.66, 95% CI 1.12–2.45), receiving neoadjuvant chemotherapy/chemoradiotherapy (OR 1.91, 95% CI 1.22–2.99; OR 1.51, 95% CI 1.17–1.96), and prolonged hospital stay (OR 1.28, 95% CI 1.02–1.60) were associated with a higher likelihood of being in moderate/severe symptom profiles, while receiving adjuvant chemotherapy or immunotherapy (OR 0.66, 95% CI 0.50–0.87) was associated with a lower likelihood.

For patients who underwent gastrectomy, subtotal (vs total) gastrectomy (OR 0.33, 95% CI 0.23–0.49) was associated with a lower likelihood of being in moderate/severe profiles, whereas female sex (OR 1.54, 95% CI 1.07–2.23) and neoadjuvant chemotherapy (OR 2.39, 95% CI 1.55–3.67) increased it (Table 2).

**Table 2:** Odds ratios (95% CI) from GLMM for baseline characteristics associated with moderate or severe symptom profiles

Variables	Esophagectomy	Gastrectomy
Intercept	0.22 (0.12–0.40)*	0.86 (0.26–2.88)
Sex		
Female vs Male	1.67 (1.30–2.14)*	1.54 (1.07–2.23)*
Age		
60–74 years vs < 60 years	0.85 (0.67–1.09)	0.71 (0.44–1.15)
≧75 years vs < 60 years	0.88 (0.61–1.26)	0.74 (0.43–1.3)
ASA classification		
ASA II vs ASA I	1.40 (0.91–2.15)	0.87 (0.28–2.68)
ASA ≧III vs ASA I	1.78 (1.12–2.83)*	1.23 (0.38–3.93)
Unknown vs ASA I	1.65 (0.82–3.30)	0.55 (0.15–1.98)
Number of comorbidities		
1 comorbidity vs No comorbidity	1.02 (0.82–1.27)	1.33 (0.88–2.01)
2 or more vs No comorbidity	1.26 (0.92–1.73)	1.09 (0.58–2.02)
Unknown vs No comorbidity	1.39 (0.76–2.53)	0.80 (0.35–1.82)
Histology		
Adenocarcinoma–diffuse vs –intestinal	1.00 (0.74–1.35)	0.90 (0.6–1.35)
Squamous cell carcinoma vs Adenocarcinoma–intestinal	1.19(0.89–1.59)	Not applicable
Other vs Adenocarcinoma–intestinal	1.37 (0.93–2.02)	2.23 (1.23–4.05)*
Unknown vs Adenocarcinoma–intestinal	1.08 (0.76–1.55)	1.10 (0.56–2.13)
cT		
cT3–4 vs cT1-2	1.05 (0.85–1.31)	1.09 (0.68–1.45)
cTX vs cT1-2	0.79 (0.44–1.41)	1.29 (0.67–2.19)
cN		
cN + vs cN0	0.97 (0.8–1.17)	0.89 (0.60–1.16)
cNX vs cN0	1.44 (0.5–4.14)	13.23 (0.76–231.97)^a^
Type of resection		
Transthoracic Ivor Lewis vs Transhiatal	1.32 (0.94–1.86)	Not applicable
Transthoracic McKeown vs Transhiatal	1.66 (1.12–2.45)*	Not applicable
Transthoracic others vs Transhiatal	1.40 (0.58–3.43)	Not applicable
Transcervical MICE vs Transhiatal	1.45 (0.65–3.24)	Not applicable
Subtotal resection vs Total resection	Not applicable	0.33 (0.23–0.49)*
Neoadjuvant chemotherapy		
Yes vs No	1.91 (1.22–2.99)*	2.39 (1.55–3.67)*
Neoadjuvant chemoradiotherapy		
Yes vs No	1.51 (1.17–1.96)*	1.59 (0.91–2.77)
Adjuvant chemotherapy or immunotherapy		
Yes vs No	0.66 (0.50–0.87)*	0.84 (0.55–1.28)
Prolonged hospital stay		
Yes vs No	1.28 (1.02–1.60)*	1.22 (0.79–1.88)
Unknown vs No	0.74 (0.46–1.20)	1.47 (0.63–3.43)

^a^Estimates for cNX in the gastrectomy subgroup may be unstable due to small sample size

*p < 0.05

## Discussion

Three symptom severity profiles were identified for patients after esophagectomy and gastrectomy: mild, moderate and severe. Most patients were in the mild profile and the fewest in the severe profile, and most stayed in the same profile or improved over time. Eating restrictions, appetite loss, dry mouth and coughing were most frequent after esophagectomy, while trouble with taste, eating restrictions and appetite loss were most common after gastrectomy. Being female and receiving neoadjuvant chemotherapy were associated with a higher likelihood of moderate to severe symptoms after undergoing esophagectomy or gastrectomy.

While most research focused on identifying symptom profiles in all symptoms [10, 21–24], our approach focused on only the gastrointestinal symptoms. This approach provided more integral gastrointestinal symptom patterns and customized information for patients in the profiles. Although the symptom patterns within the profiles varied, they generally formed centered polygon shapes with a clear hierarchy of severity (i.e. mild, moderate, severe), as shown in the radar charts. The results indicated that common gastrointestinal symptoms occurred similarly at certain periods for patients who underwent gastrectomy or esophagectomy, giving insights into symptom management for patients at different stages of their treatment.

The overall performance of clustering evaluated by Silhouette scores ranged between 0.12–0.36 and were considered low. These findings indicate relatively weak cluster separation and support interpreting the clustering analysis as exploratory. One study that evaluated different clustering methods of cancer symptoms found similar results, where the Silhouette scores of symptom clustering by multiple algorithms, including K-means, were with similar Silhouette scores between 0.05–0.19 [25]. The result highlighted the challenges of clustering clinical data, as most of the features of these data are normally highly correlated. To tackle the correlated features and enhance the performance of clustering, some studies would apply principal component analysis (PCA) on the cancer symptoms for dimension reduction [23, 24, 26–28], thus decreasing the noise. However, PCA compresses features into abstract components, mixing symptom information together and obscuring direct clinical interpretation. Therefore, we did not incorporate PCA in our study.

Moreover, most patients did not shift between different profiles over time. Since having multiple gastrointestinal symptoms was negatively associated with quality-of-life outcomes in previous research [29], our results indicated that more focus on these severe symptom profiles, even before the resection, could potentially be crucial for improving the patients’ post-resection outcomes. The clustering results can assist clinicians in clearer communication of expected symptom trajectories after the surgery, building realistic expectations and encouraging early reporting of severe symptoms for the patients.

Follow-up care after esophagectomy or gastrectomy aimed at gastrointestinal symptom management was not fully standardized in the Netherlands. Existing guidelines address several individual symptoms, as some hospitals have introduced multidisciplinary protocols to streamline care [30, 31]. However, these approaches still largely focus on single symptoms, even though patients experience multiple, co-occurring problems [4]. By targeting symptom profiles rather than individual symptoms, treatment strategies could become more effective and foster better communication between clinicians and patients [32]. Patients clustered with higher severity might need earlier multidisciplinary support, such as nutritional and rehabilitation care, and could benefit from more targeted monitoring and intervention [33].

In this study, the longitudinal data allowed us to track how the symptom profile patterns change over time, together with the shifting of the patients’ clusters. We conducted separate clustering at different assessment periods instead of restricting the patients to only one profile throughout all periods, which provided a more flexible approach to reveal the clustering of the time-dependent symptom changes of patients who underwent esophagectomy or gastrectomy. However, this approach also limits direct comparability of profile membership across time because profile centroids may differ between assessment periods. Consequently, transitions between profiles should not be interpreted as absolute changes in symptom severity.

Several additional limitations should be considered. The EORTC QLQ-C30 and QLQ-OG25 are validated tools for assessing health-related quality of life in patients with oesophageal or gastric cancer [34], but they summarize symptoms over the preceding week and may not fully capture day-to-day variability in symptoms, intermittent severe symptoms and lifestyle adaptations. Furthermore, lower gastrointestinal symptoms were underrepresented, as the available questionnaires included only diarrhea and constipation. Although baseline symptom burden was incorporated into the longitudinal analysis, longstanding habitual symptoms could not be distinguished from cancer- or treatment-related symptoms. We only included patients who completed at least two questionnaires, which ensured that at least one follow-up per patient was included in the analysis. However, the number of responses per patient varied considerably, which may have influenced the stability of the clustering results and the observed transition patterns. Selection bias possibly occurred as patients who complete questionnaires are usually younger, less frail, and have a better prognosis [35], which can lead to the underestimation of the symptom outcomes, with fewer patients being clustered in moderate or severe symptom profiles. Meanwhile, some missing baseline characteristics of the patients were encoded as “unknown”, which cannot give any valuable interpretation. Additionally, even though we included the factor of prolonged hospital stay, we did not have information on postoperative complication, as well as recurrence of disease or death, which might have resulted in dropouts.

In conclusion, this exploratory longitudinal analysis identified stable gastrointestinal symptom severity profiles during the first postoperative year following esophagectomy or gastrectomy. The persistence of profile membership over time suggests that baseline symptom burden may be informative for identifying patients at risk for ongoing moderate-to-severe gastrointestinal symptoms. These findings support the potential value of early risk-stratified monitoring and targeted symptom management in survivorship care. Future studies using alternative longitudinal modeling approaches and more comprehensive data collection are warranted to confirm and refine these patterns before clinical implementation.

## Supplementary Information

Below is the link to the electronic supplementary material.

Supplementary file1 (PDF 705 KB)

## Data Availability

The dataset supporting the conclusion of this article was used under license and so are not publicly available. Data is available via the NCR ([https://iknl.nl/] (https:/iknl.nl)) upon request and after approval by the NCR. The plan for the statistical analysis will be made available by the corresponding author upon request.
