# Supplementary material for: Gastrointestinal symptom profiles after resection of esophageal or gastric cancer
Source: Support Care Cancer. 2026 Oct 7;34(10):1069. doi: 10.1007/s00520-026-11293-7 (PMC13645954; doi:10.1007/s00520-026-11293-7)
Supplement: Supplementary file 1 [file 520_2026_11293_MOESM1_ESM.pdf]

Gastrointestinal Symptom Profiles After Resection of Esophageal or Gastric Cancer

Supportive Care in Cancer

Yipei Lee[1,2,3], Dimitris Katsimpokis[1,9], Felice N. van Erning[1,6], Grard A.P. Nieuwenhuijzen[6], Hanneke W.M. van Laarhoven[2,3], Suzanne S. Gisbertz[3,4], Bastiaan R. Klarenbeek[5], Paul M. Jeene[7], Roos E. Pouw[8], Loes Noteboom[4], Rob H.A. Verhoeven[1,2,3], Pauline A.J. Vissers[1,5]

[1]Department of Research and Development, Netherlands Comprehensive Cancer Organization (IKNL),
Rijnkade 5, 3511 LC Utrecht, The Netherlands.
[2]Amsterdam UMC location University of Amsterdam, Department of Medical Oncology,
De Boelelaan 1117, 1081 HV Amsterdam, The Netherlands.
[3]Cancer Center Amsterdam, Cancer Treatment and Quality of Life,
De Boelelaan 1117, 1081 HV Amsterdam, The Netherlands.
[4]Amsterdam UMC location University of Amsterdam, Department of Surgery,
De Boelelaan 1117, 1081 HV Amsterdam, The Netherlands.
[5]Department of Surgery, Radboud University Medical Center,
Geert Grooteplein Zuid 10, 6525 GA Nijmegen, The Netherlands.
[6]Department of Surgery, Catharina Hospital,
Michelangelolaan 2, 5623 EJ Eindhoven, The Netherlands.
[7] Radiotherapiegroep,
Nico Bolkesteinlaan 85, 7416 SE Deventer, The Netherlands.
[8]Department of Gastroenterology and Hepatology, University Medical Center Utrecht,
Heidelberglaan 100, 3584 CX Utrecht, The Netherlands.
[9]Department of Medical Informatics, Amsterdam UMC, Amsterdam, The Netherlands.

Corresponding Author:
Yipei Lee
Address: De Boelelaan 1117, 1081 HV Amsterdam, The Netherlands.
E-Mail: y.lee@amsterdamumc.nl

(a) Esophagectomy — Baseline

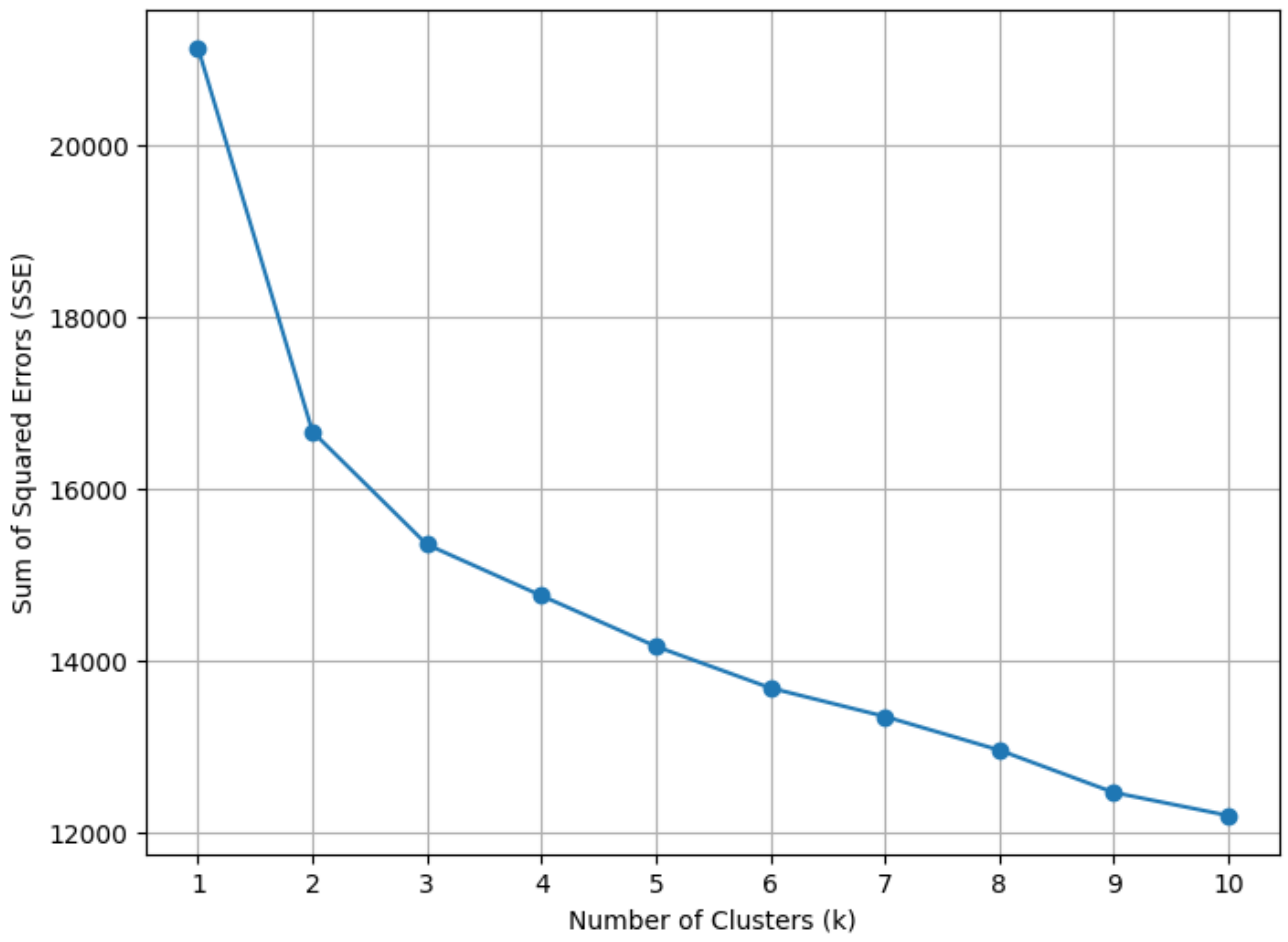

(b) Esophagectomy — 0–3 months

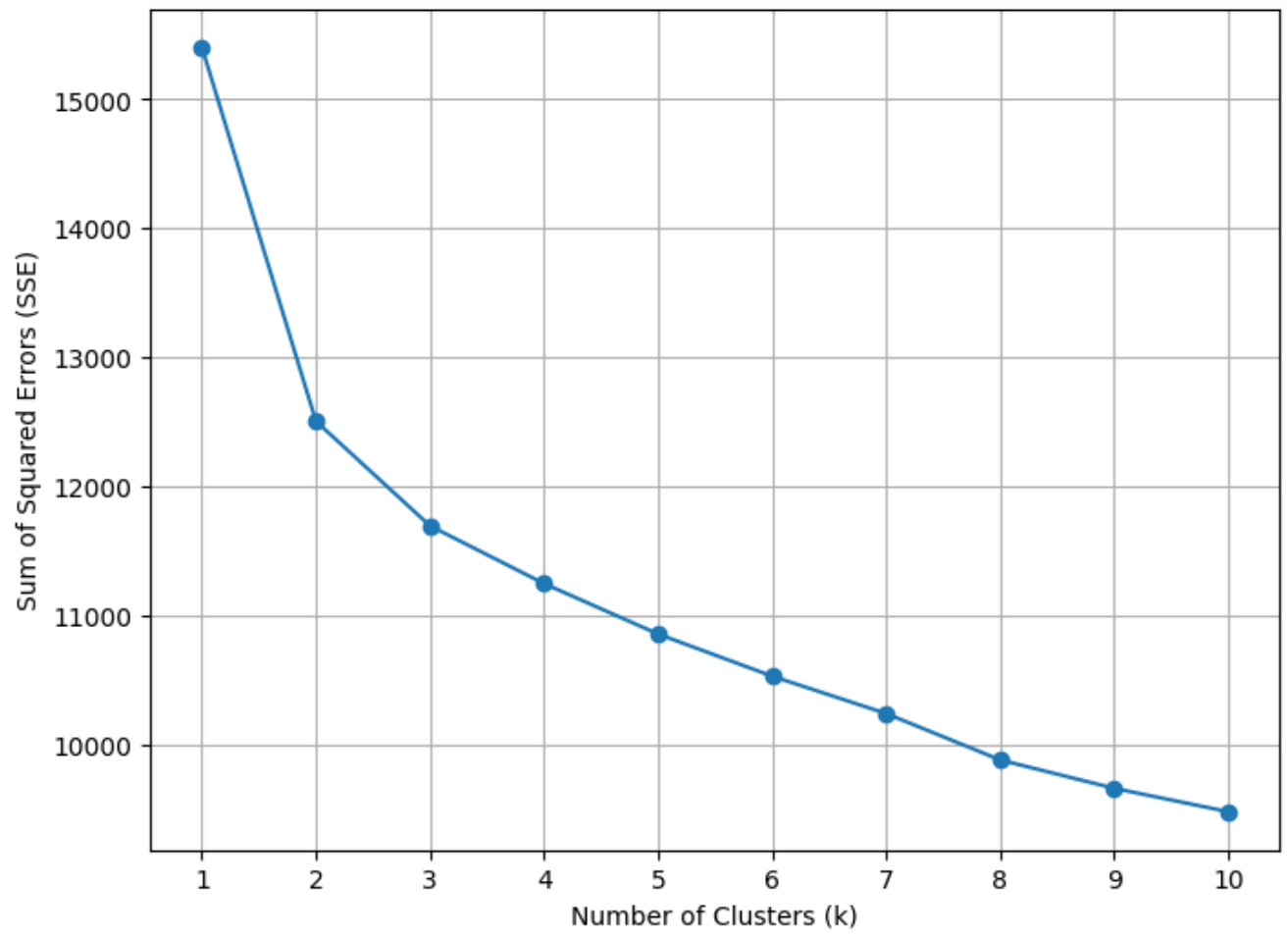

(c) Esophagectomy — 3–6 months

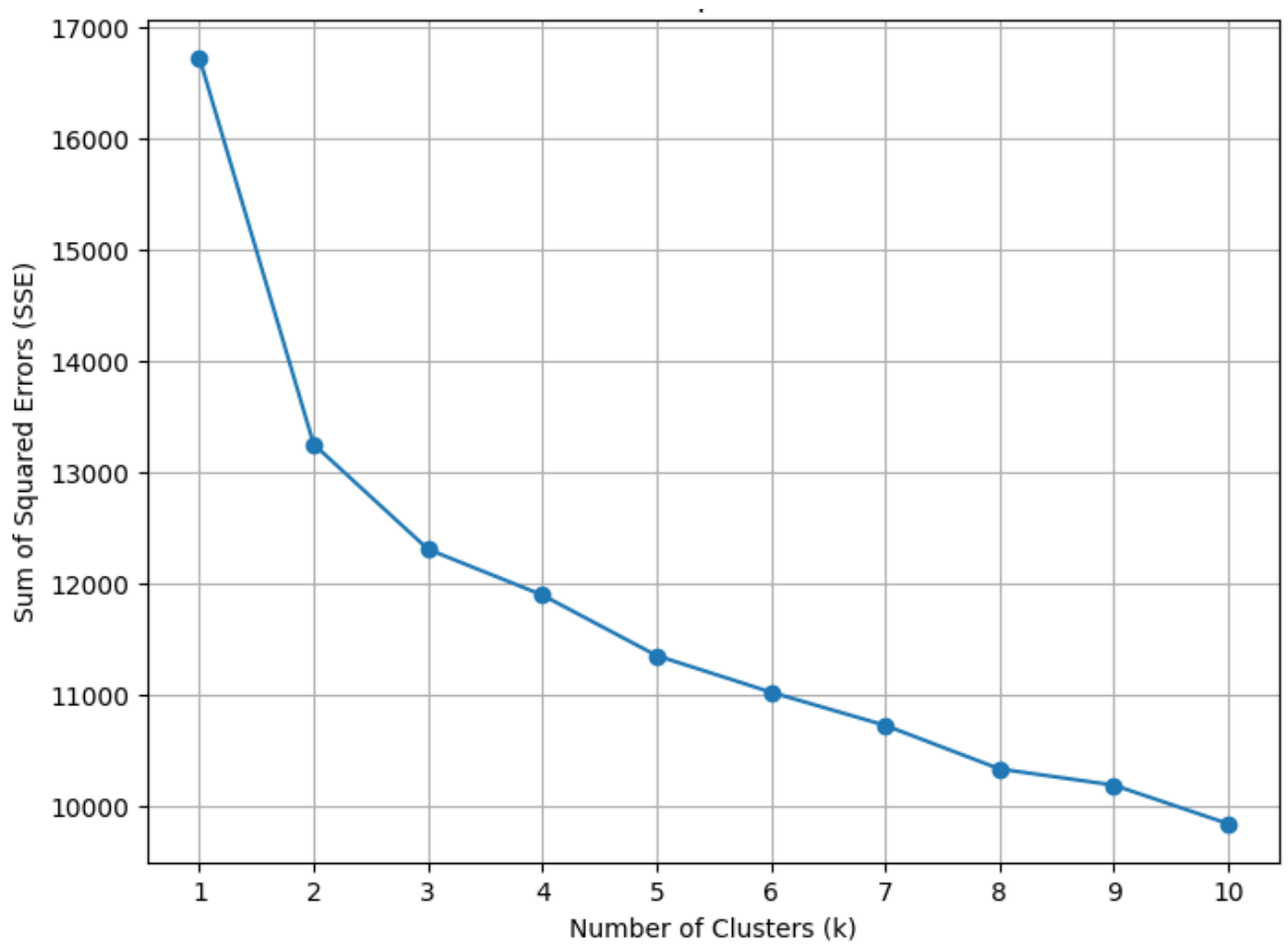

(d) Esophagectomy — 6–9 months

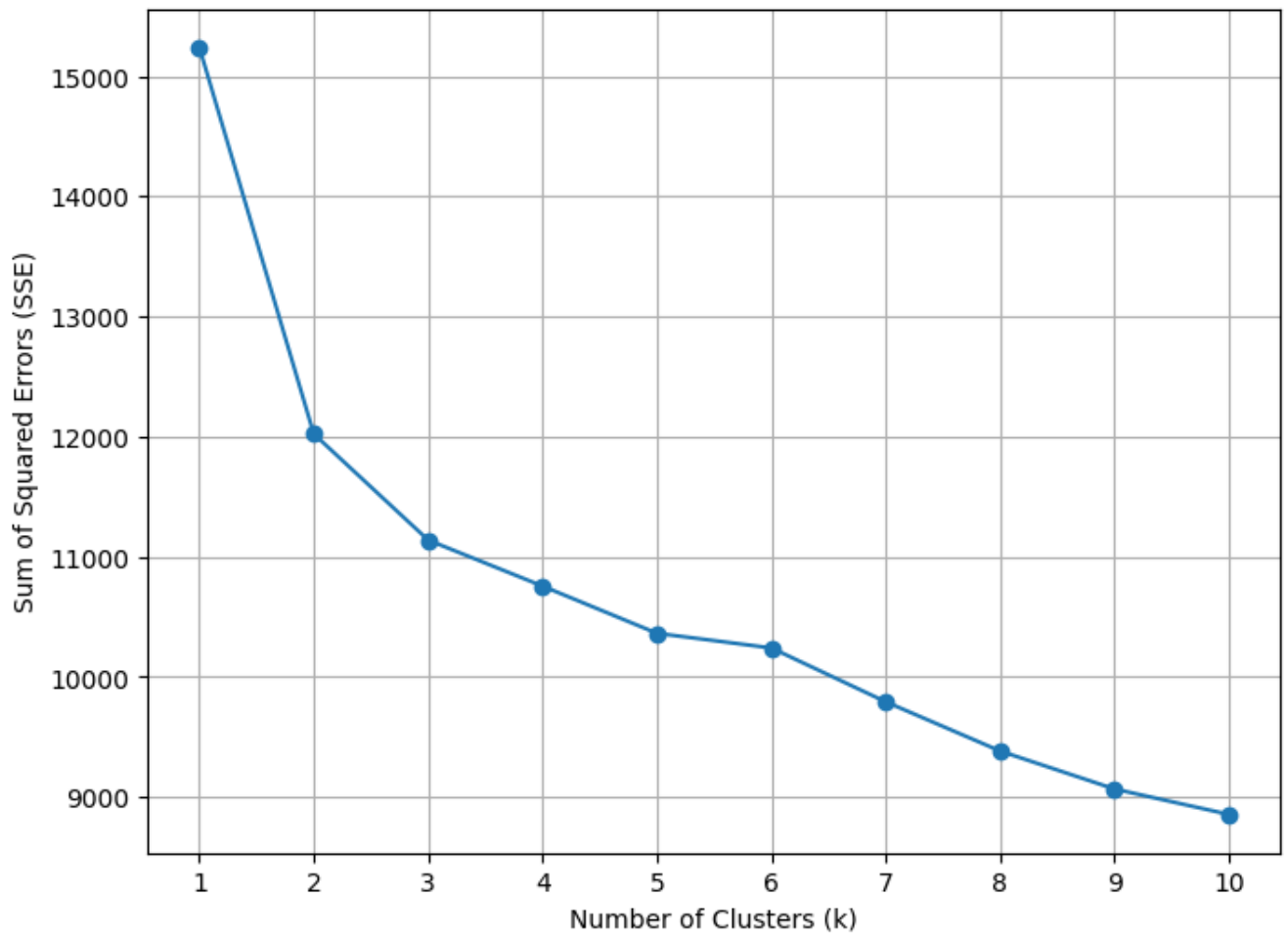

(e) Esophagectomy — 9–12 months

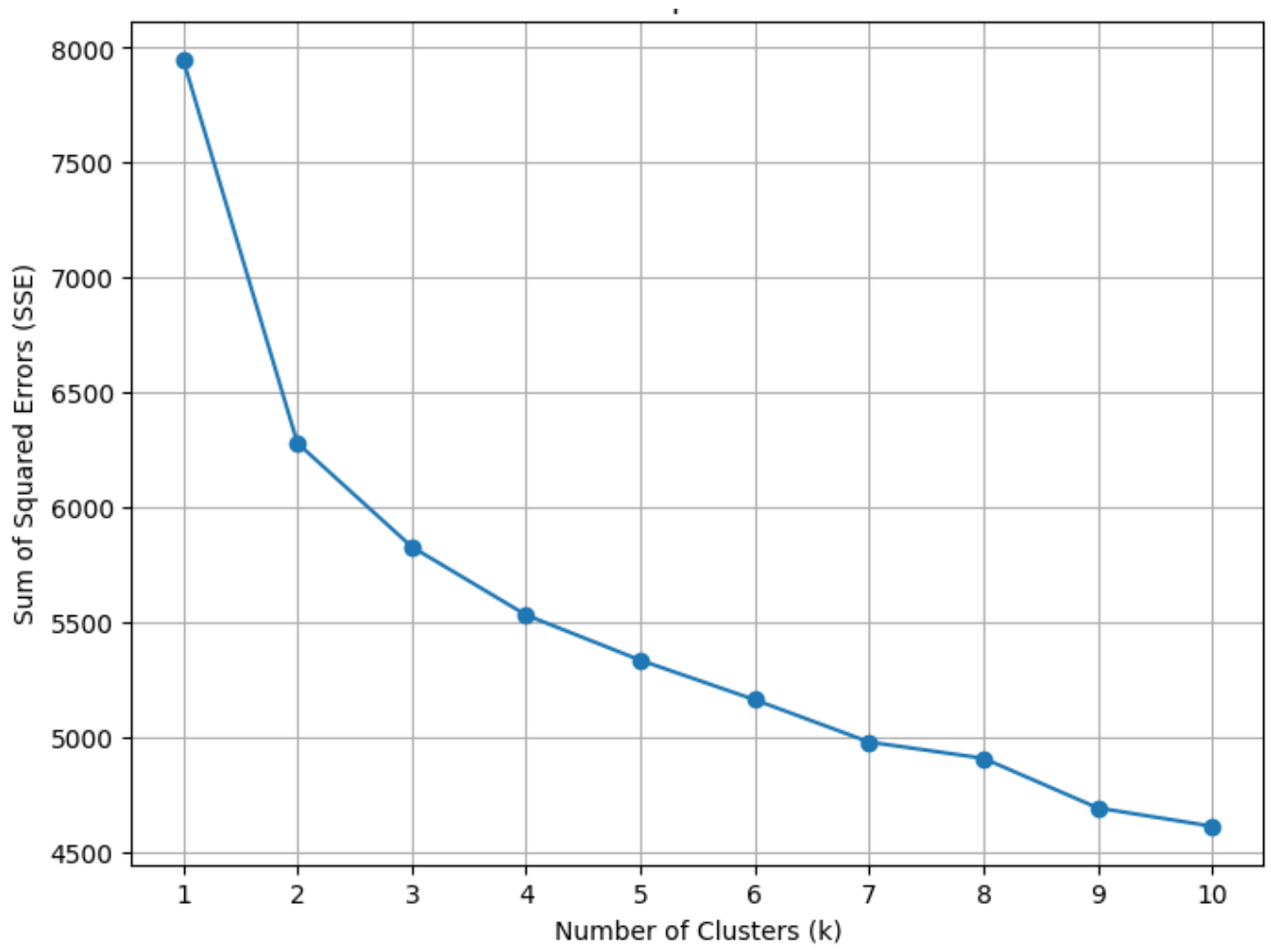

(f) Gastrectomy — Baseline

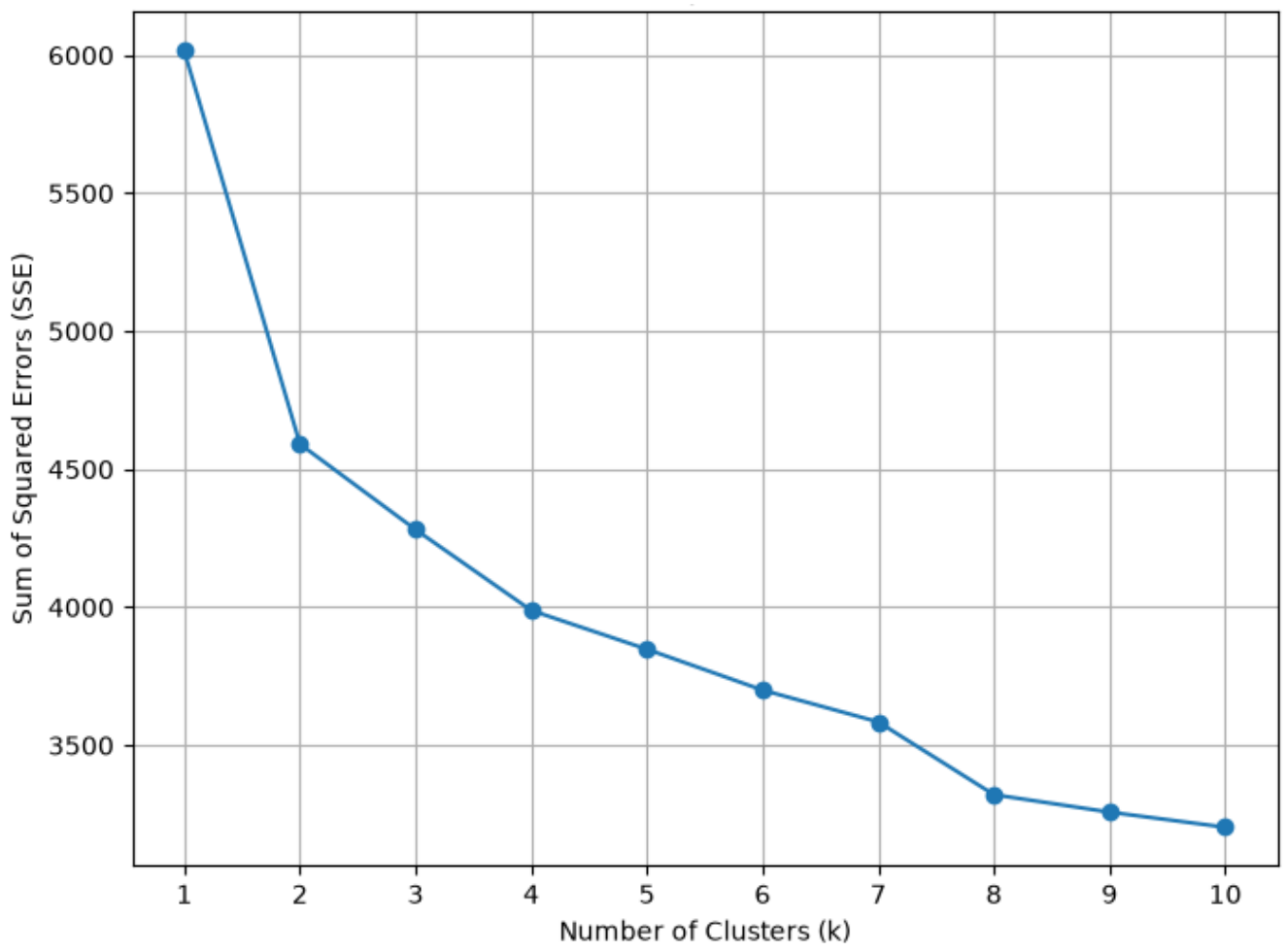

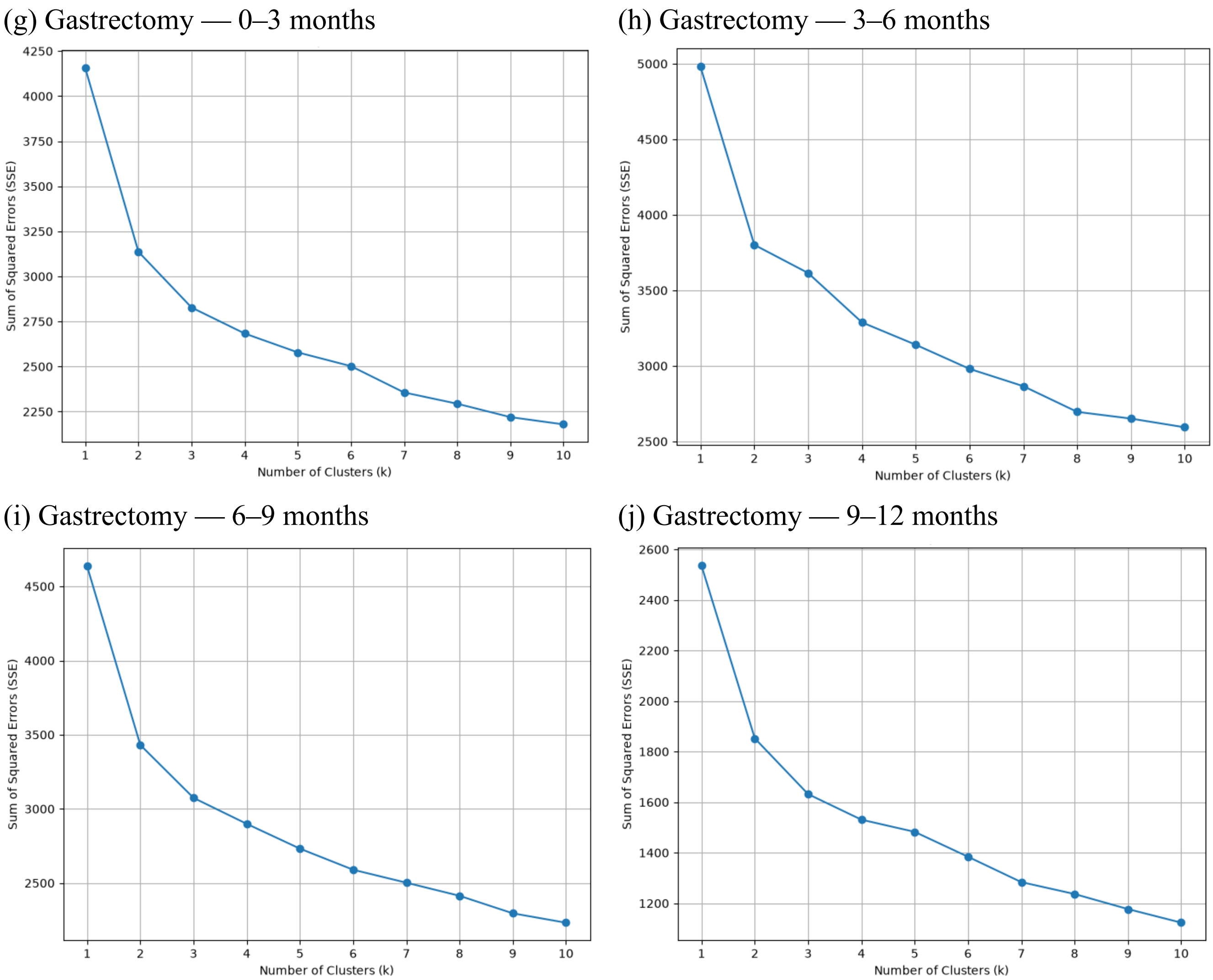

Supplementary Figure 1. Elbow plots for selection of the number of K-means clusters across assessment periods.

Elbow plots are shown separately for patients who underwent esophagectomy and gastrectomy at baseline, 0–3 months, 3–6 months, 6–9 months, and 9–12 months after resection. The x-axis represents the number of clusters (k = 1–10), and the y-axis represents the within-cluster sum of squared errors (SSE). The three-profile solution (k = 3) was selected based on the Elbow method together with interpretability of the resulting patient symptom profiles.

(a) Esophagectomy —mean symptom scores and SDs for each symptom profile over time

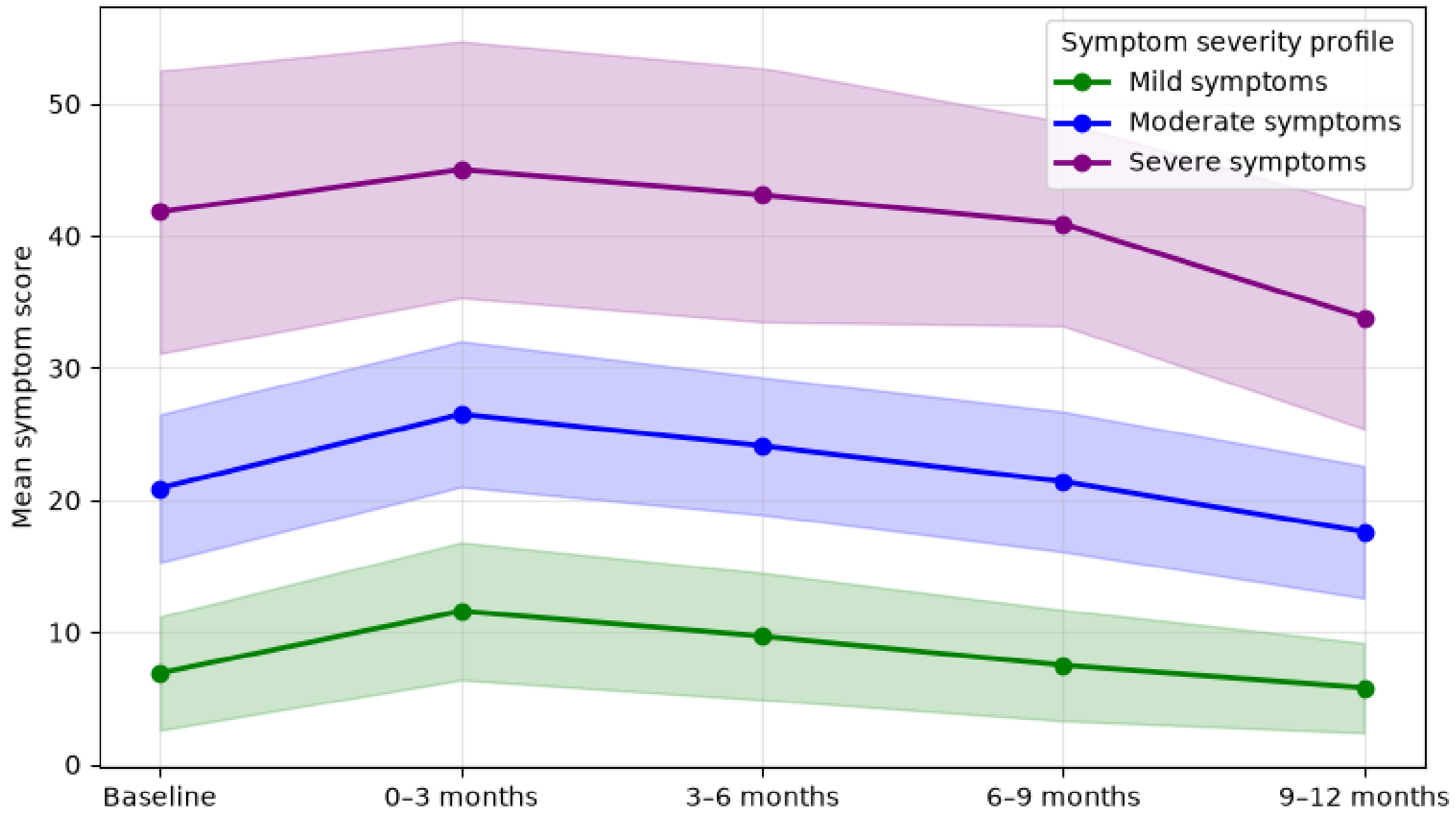

(b) Gastrectomy —mean symptom scores and SDs for each symptom profile over time

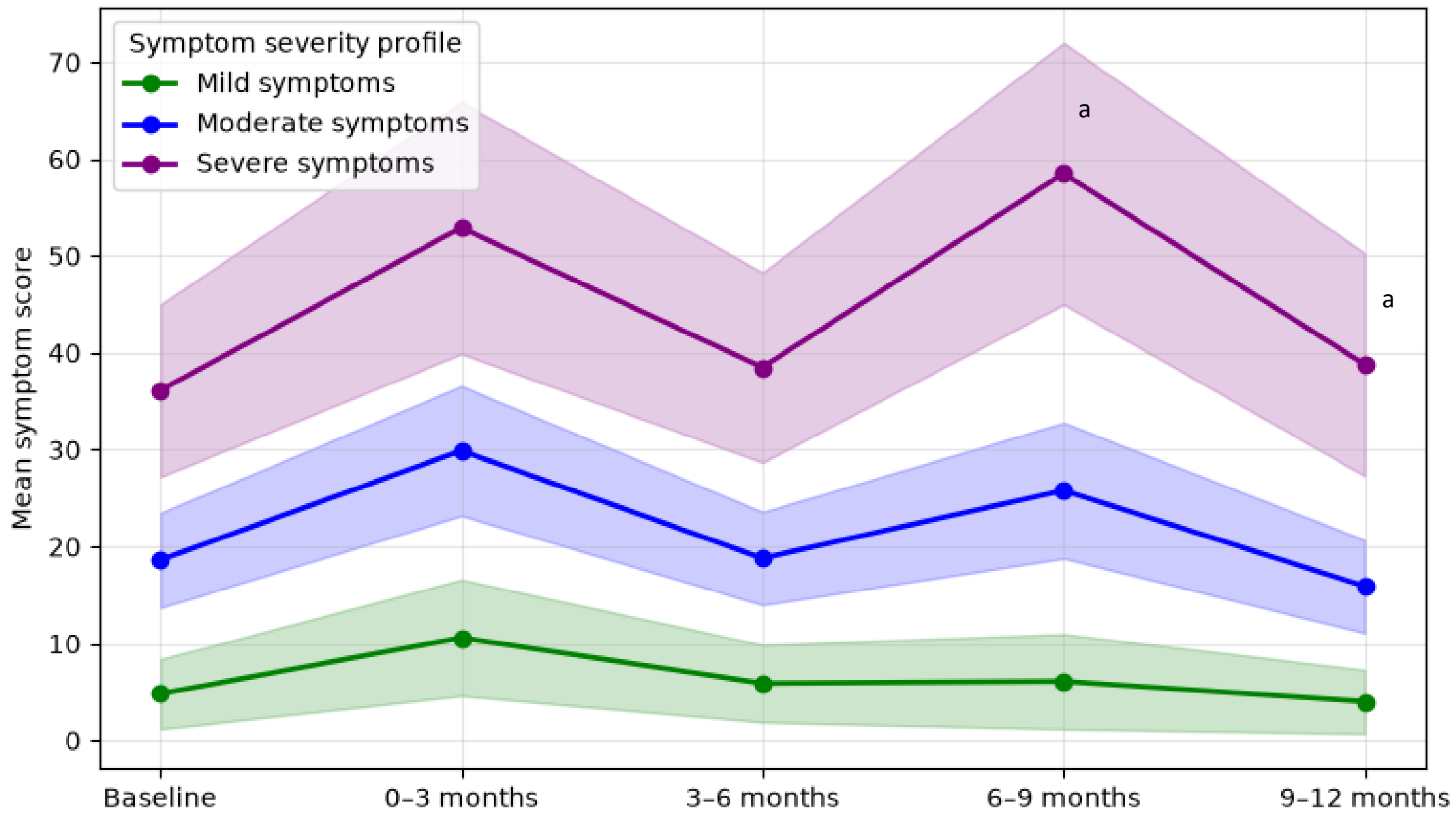

Supplementary Figure 2. Mean symptom scores over time by symptom severity profile. Mean symptom scores are shown across assessment periods for patients undergoing (a) esophagectomy and (b) gastrectomy. Solid lines represent the mean symptom score for each profile, and shaded areas represent ±1 standard deviation (SD). Green, blue, and purple indicate the mild, moderate, and severe symptom profiles, respectively.

[a] Estimates for the severe symptom profiles at 6–9 and 9–12 months after gastrectomy should be interpreted with caution due to the small sample size (n < 20).

Supplementary Table 1 Overview of the questions included in the items and scales for gastrointestinal symptoms, health-related quality of life and functioning

Gastrointestinal symptoms	
**EORTC QLQ-C30**	**Nausea/vomiting**<br>1. During the past week, have you felt nauseated?<br>2. During the past week, have you vomited?<br><br>**Constipation**<br>1. During the past week, have you been constipated?<br><br>**Diarrhea**<br>1. During the past week, have you had diarrhea?<br><br>**Appetite loss**<br>1. During the past week, have you lacked appetite?
**EORTC QLQ-OG25**	**Dysphagia**<br>1. During the past week, have you had problems eating solid foods?<br>2. During the past week, have you had problems eating liquidized or soft foods?<br>3. During the past week, have you had problems drinking liquids?<br><br>**Eating restrictions**<br>1. During the past week, have you had trouble enjoying your meals?<br>2. During the past week, have you felt full up too quickly after beginning to eat?<br>3. During the past week, has it taken you a long time to complete your meals?<br>4. During the past week, have you had difficulty eating?<br><br>**Reflux**<br>1. During the past week, have you had acid indigestion or heartburn?<br>2. During the past week, has acid or bile coming into your mouth been a problem?<br><br>**Odynophagia**<br>1. During the past week, have you had discomfort when eating?<br>2. During the past week, have you had pain when you eat?<br><br>**Pain and discomfort**<br>1. During the past week, have you had pain in your stomach area?<br>2. During the past week, have you had discomfort in your stomach area?<br><br>**Dry mouth**<br>1. During the past week, have you had a dry mouth?<br><br>**Choked when swallowing**<br>1. During the past week, have you choked when swallowing?<br><br>**Trouble with taste**<br>1. During the past week, have you had problems with your sense of taste?<br><br>**Coughing**<br>1. During the past week, have you coughed?<br><br>**Swallowing saliva**<br>1. During the past week, have you had difficulty swallowing your saliva?

Talking
1. During the past week, have you had difficulty talking?

Supplementary Table 2 Clustering result in different time periods

	N (%)	Mean score	N (%)	Mean score	N (%)	Mean score	N (%)	Mean score	N (%)	Mean score
Time	Baseline (before resection)		0~3 months after resection		3~6 months after resection		6~9 months after resection		9~12 months after resection	
Esophagectomy										
Silhouette Score	0.21		0.12		0.18		0.21		0.19	
mild	814 (58.3%)	6.9	436 (42.5%)	11.6	643 (57.8%)	9.7	613 (60.5%)	7.5	295 (55.9%)	5.8
moderate	455 (32.6%)	20.9	432 (42.1%)	26.5	353 (31.7%)	24.1	319 (31.5%)	21.4	169 (32.0%)	17.6
severe	127 (9.1%)	41.8	158 (15.4%)	45.0	116 (10.4%)	43.1	81 (8.0%)	40.9	64 (12.1%)	33.8
Gastrectomy										
Silhouette Score	0.22		0.23		0.19		0.36		0.24	
mild	224 (55.9%)	4.8	167 (60.3%)	10.6	179 (53.9%)	5.9	227 (73.5%)	6.1	96 (56.8%)	4.0
moderate	114 (28.4%)	18.6	86 (31.0%)	29.9	102 (30.7%)	18.9	75 (24.3%)	25.8	55 (32.5%)	16.0
severe	63 (15.7%)	36.1	24 (8.7%)	52.9	51 (15.4%)	38.5	7 (2.3%)	58.5 [a]	18 (10.7%)	38.8 [a]

[a] Estimates for the severe symptom profiles at 6–9 and 9–12 months after gastrectomy should be interpreted with caution due to the small sample size (n < 20).
